# A novel marine nitrite-oxidizing *Nitrospira* species from Dutch coastal North Sea water

**DOI:** 10.3389/fmicb.2013.00060

**Published:** 2013-03-18

**Authors:** Suzanne C. M. Haaijer, Ke Ji, Laura van Niftrik, Alexander Hoischen, Daan Speth, Mike S. M. Jetten, Jaap S. Sinninghe Damsté, Huub J. M. Op den Camp

**Affiliations:** ^1^Department of Microbiology, Institute for Water and Wetland Research, Radboud University NijmegenNijmegen, Netherlands; ^2^Department of Human Genetics, Nijmegen Center for Molecular Life Sciences, Institute for Genetic and Metabolic Disease, Radboud University NijmegenNijmegen, Netherlands; ^3^Department of Marine Organic Biogeochemistry, Royal Netherlands Institute for Sea ResearchDen Burg, Texel, Netherlands

**Keywords:** marine nitrification, enrichment, *Nitrosomonas*, *Nitrospira*, fluorescence *in situ *hybridization, transmission electron microscopy, 16S rRNA

## Abstract

Marine microorganisms are important for the global nitrogen cycle, but marine nitrifiers, especially aerobic nitrite oxidizers, remain largely unexplored. To increase the number of cultured representatives of marine nitrite-oxidizing bacteria (NOB), a bioreactor cultivation approach was adopted to first enrich nitrifiers and ultimately nitrite oxidizers from Dutch coastal North Sea water. With solely ammonia as the substrate an active nitrifying community consisting of novel marine *Nitrosomonas *aerobic ammonia oxidizers (ammonia-oxidizing bacteria) and *Nitrospina *and *Nitrospira *NOB was obtained which converted a maximum of 2 mmol of ammonia per liter per day. Switching the feed of the culture to nitrite as a sole substrate resulted in a *Nitrospira* NOB dominated community (approximately 80% of the total microbial community based on fluorescence *in situ* hybridization and metagenomic data) converting a maximum of 3 mmol of nitrite per liter per day. Phylogenetic analyses based on the 16S rRNA gene indicated that the *Nitrospira* enriched from the North Sea is a novel *Nitrospira* species with *Nitrospira marina* as the next taxonomically described relative (94% 16S rRNA sequence identity). Transmission electron microscopy analysis revealed a cell plan typical for *Nitrospira* species. The cytoplasm contained electron light particles that might represent glycogen storage. A large periplasmic space was present which was filled with electron dense particles. *Nitrospira*-targeted polymerase chain reaction analyses demonstrated the presence of the enriched *Nitrospira *species in a time series of North Sea genomic DNA samples. The availability of this new *Nitrospira *species enrichment culture facilitates further in-depth studies such as determination of physiological constraints, and comparison to other NOB species.

## INTRODUCTION

The ocean is the largest reservoir of fixed nitrogen on Earth containing about five times more fixed nitrogen than terrestrial systems (Gruber, 2008) which renders marine systems of major importance to global nitrogen cycling. Nitrogen, in the bioavailable forms of ammonium and nitrate, is one of the key nutrients in marine waters and may limit primary production especially in coastal systems (Downing, 1997; Wollast, 1998; Zehr and Kudela, 2011). Most of the fixed organic nitrogen in the ocean is converted to nitrate by remineralization consisting of ammonification and nitrification (Gruber, 2008). In the two-step process of nitrification, ammonia is oxidized first to nitrite by aerobic ammonia-oxidizing microorganisms and then to nitrate by aerobic nitrite-oxidizing microorganisms.

The microbial mediators of nitrification have intrigued scientists ever since the hallmark publication by Winogradsky (1890) in which not only the ability of nitrifying organisms to withdraw energy from mineral substances was reported but it was also concluded that these microorganisms assimilate carbon from carbon dioxide. At present, after 120 years of research efforts, many nitrifying microorganisms are available in culture, and therefore amenable to physiological characterization, but marine species are underrepresented. For instance, when evaluating the phylogeny of β-proteobacterial ammonia-oxidizing bacteria (AOB) Aakra et al. (2001) examined no less than 38 isolates of which only five had a marine origin. In the review by Koops and Pommerening-Röser (2001) on the distribution and ecophysiology of nitrifying bacteria the phylogenetic relationship of 19 cultured AOB species is shown and for only five of those species a preference for a marine habitat is indicated. In marine ecosystems ammonia-oxidizing archaea (AOA) species have been shown to outnumber their bacterial counterparts based on direct cell counts and gene (16S rRNA and *amo*A) copy numbers (Francis et al., 2005; Wuchter et al., 2006; Mincer et al., 2007). Currently, however, the only two cultured AOA species with a marine or estuarine origin are *Nitrosopumilus maritimus* (Könneke et al., 2005; Wuchter et al., 2006; Park et al., 2010; Walker et al., 2010) and *Nitrosoarchaeum limnia *(Blainey et al., 2011; Mosier et al., 2012).

Marine species have been found in four of the recognized nitrite-oxidizing bacterial genera (*Nitrospira*, *Nitrospina*, *Nitrobacter*, and *Nitrococcus*; Ward and Carlucci, 1985). For the genus *Nitrotoga* (Alawi et al., 2007) no marine species are presently known. The recently described nitrite oxidizer *Nitrolancetus hollandicus *(Sorokin et al., 2012), which in contrast to the previously known proteobacterial nitrite oxidizers belongs to the *Chloroflexi* phylum, was isolated from a reactor treating sewage plant digester effluent and no data on its salt tolerance or environmental distribution is yet available.

Within the process of nitrification ammonia oxidation to nitrite is the rate-limiting step and nitrite rarely accumulates in the process of nitrification (Philips et al., 2002; Arp, 2009). This may explain why nitrite-oxidizing bacteria (NOB) are often overlooked in marine environmental studies concerning nitrification. Recent findings in the Namibian oxygen minimum zone (OMZ) by Füssel et al. (2011), however, indicate that nitrite oxidation rates may even exceed ammonia oxidation rates. Marine *Nitrospira *species have been isolated from a surface water sample of the Gulf of Maine (Watson et al., 1986) as well as from marine recirculation aquaculture system biofilters (Keuter et al., 2011; Brown et al., 2013). In addition they have been described as inhabitants of marine sponges (Hoffmann et al., 2009; Off et al., 2010). *Nitrospina *species have been detected in both coastal and open ocean habitats (Suzuki et al., 2004; DeLong et al., 2006; Beman et al., 2010) based on 16S rRNA gene sequences. Moreover, co-variation of archaeal *amo*A and 16S rRNA genes with *Nitrospina*-like 16S rRNA genes has been observed which suggests that *Nitrospina* NOB may be natural nitrite-oxidizing partners of marine AOA (Mincer et al., 2007; Santoro et al., 2010). Fluorescence *in situ* hybridization (FISH) analyses using probes targeting all nitrite-oxidizing genera known at that time by Füssel et al. (2011) on Namibian OMZ samples demonstrated the presence of only *Nitrospina* and *Nitrococcus* NOB in equal abundance.

The elucidation of the ecophysiology of marine nitrite oxidizers in part is complicated by the difficulties in combining molecular data (e.g., presence and abundance of particular genes or species) with cultivation-derived parameters (e.g., proof of physiological capabilities, affinities, growth rates, salt tolerance). Increasing the availability of cultured species and ultimately determining their key physiological traits is helpful because it will aid in designing directed environmental research. Knowledge of physiological constraints of different strains and species, for instance, enables making informed guesses about which particular strain or species inhabits a certain habitat. To increase the number of cultivated marine nitrite oxidizers, a bioreactor set-up was used in the present study to first enrich a marine assemblage of aerobic ammonia oxidizers and nitrite oxidizers and ultimately solely the nitrite oxidizers from North Sea coastal water. The microbial community composition was evaluated by FISH analyses and the phylogenetic position of the enriched aerobic ammonia oxidizers and nitrite oxidizers determined by 16S rRNA gene sequence [polymerase chain reaction (PCR) and metagenome data] based analyses. The cell plan of the enriched nitrite-oxidizing *Nitrospira* species was visualized with transmission electron microscopy (TEM) and, using a newly designed primer pair targeting *Nitrospira *species, its presence was detected in a time series (Wuchter et al., 2006; Pitcher et al., 2011) of high molecular weight DNA isolated from the same coastal sampling site.

## MATERIALS AND METHODS

### INOCULUM DESCRIPTION AND REACTOR SET-UP

Water representative of Dutch coastal North Sea water (Pitcher et al., 2011) was collected in February 2007 at high tide at the jetty of the Royal Netherlands Institute for Sea Research situated on the island Texel (53°00′25′N, 4°78′27′E). An aliquot of 48 L North Sea water was filtered using a HF80S polysulfone capillary artificial Kidney/Hemofilter (Fresenius Medical Care Nederland BV, Nieuwkuijk, the Netherlands). This resulted in 2 L of 24-fold concentrated biomass suspension and a cleared solution (filtrate) devoid of particles. The collected biomass suspension was incubated in a sterile glass and stainless steel reactor (adaptive, 2 L working volume). Heat-sterilized (20 min, 120°C, 15 kPa) filtrate supplemented with ammonium or nitrite (from 1 M sterile stocks of NH_4_Cl and NaNO_2_) was used as a medium. Oxygen and pH were monitored online using Applikon (Applikon Biotechnology BV, Schiedam, the Netherlands) sensors. Nitrite concentrations were determined offline in liquid samples withdrawn daily from the reactor using Merckoquant test strips (Merck BV, Schiphol-Rijk, the Netherlands) and ammonium as well as nitrite concentration weekly using colorimetric methods (see below). The reactor was kept at a pH 7.8 with solutions of sterile 1 M NaHCO_3_ and 0.6 M HCl, operated at room temperature 22 ± 2°C, stirred at 150 rpm, and supplied with an air flow of 80 ml/min.

### ENRICHMENT WITH AMMONIA AS THE SUBSTRATE

The culture was amended with 500 μM NH_4_Cl and incubated for 19 days as a batch and then for another 2 days after addition of 400 μM NH_4_Cl. To avoid nitrite toxicity, the reactor system was thereafter switched to a continuous mode of operation using medium containing 750 μM ${NH}_{4}^{+}$ at a dilution rate of 0.25 day^-1^. When nitrite disappeared from the culture, indicating activity of nitrite oxidizers, the ammonium concentration was subsequently increased to 1.5 mM after 3 months, and further to 2, 3, and finally 10 mM after 4, 4.5, and 5.5 months, respectively. The reactor was switched to nitrite as the sole substrate after 7 months.

### ENRICHMENT WITH NITRITE AS THE SUBSTRATE

To stimulate growth of the nitrite oxidizers, a batch mode of operation was adopted and 750 μM NaNO_2_ provided as the substrate. Whenever nitrite was depleted, it was restored to 750 μM. In this manner, a total of 43 mmol of nitrite were supplied in the first month of operation with nitrite as the sole substrate. Wall growth was suspended and the biomass diluted fourfold by replacement of reactor content with medium respectively, 1 week and 1 month after the switch to nitrite. After 1 month, a fed-batch mode of operation was adopted by adding medium containing 10 mM NaNO_2_ at a flow rate starting at 40 ml per day. The pump rate of the influent was increased manually in small (~10 ml day^-1^) steps whenever ${NO}_{2}^{-}$ levels remained below 2 mg/L to a final rate of 100 ml per day. To retain biomass, the reactor content was allowed to settle once a week for at least 1 h after which clarified liquid was removed to maintain a maximum reactor volume of 2 L. Removal of wall growth and fourfold dilution of the biomass were performed as described above after 2 and 4 months. The influent nitrite concentration was raised from 10 to 20, 40, 60, 80 mM and finally 100 mM after 6, 9, 10, 10.5, and 11 months, respectively, by increasing the flow rate from 40 to 100 ml per day in 10 ml steps keeping ${NO}_{2}^{-}$ levels below 2 mg/L. In order to prevent suboptimal nitrite oxidizer growth rates due to carbon limitation, the gas flow of 80 ml/min of air was supplemented with 10 ml/min Argon/CO_2_ (95%/5%) from 7.5 months onward. It has furthermore been reported that iron as well as phosphate may become limiting compounds for growth (van de Vossenberg et al., 2008) when a medium containing only natural sea salts is used to enrich marine microorganisms. Therefore, the influent was supplemented with 0.261 ml/L 1 M KH_2_PO_4_ and 0.45 ml/L of a 5 g/L FeSO_4_·7H_2_O + 5 g/L ethylenediaminetetraacetic acid (EDTA) titriplex 3 solution from month eight onward. The reactor was operated for 12 months with nitrite as the sole substrate. During the last month the biomass was no longer allowed to settle prior to medium replenishment resulting in an actual dilution rate of 0.05 day^-1^.

### CHEMICAL ANALYSES

To estimate nitrite concentrations liquid samples were measured directly using Merckoquant^®^ teststrips (range for nitrate 10–500 mg/L; nitrite 2–80 mg/L, Merck BV, Schiphol-Rijk, the Netherlands). At least once a week, 0.5 ml aliquots were centrifuged (5 min 10,000 × *g*) and the resulting supernatants used for more elaborate colorimetric analyses to monitor residual ammonium and nitrite concentrations. To measure nitrite, a colorimetric method adapted from Griess-Romijn-van Eck (1966) was used. A mixture of 50 μl with 0.5 ml of reagent A (10 *g* of sulfanilic acid in 1 L 1 M HCl) and 0.5 ml reagent B (1 **g** 1-*N*-napthylethylenediamine dihydrochloride in 1 L distilled water) was incubated for 10 min at room temperature, and measurements performed at 540 nm. Ammonium concentrations were determined using ortho-phtaldialdehyde (OPA) reagent (Roth, 1971; Taylor et al., 1974). The OPA reagent consisted of 0.54 *g* of OPA dissolved in 10 ml of absolute ethanol, with 50 μl of β-mercaptoethanol, and filled to 100 ml with sodium phosphate buffer (0.3 M pH 7.3). To measure ammonium concentrations between 0.25 and 5 mM 50 μl sample was mixed with 800 μl OPA reagent, incubated (20 min, room temperature, in the dark), and the extinction measured (420 nm). To measure in the range of 5–300 μM, 100 μl of sample was mixed with 2 ml OPA reagent containing only 0.054 g/100 ml OPA, incubated (20 min, room temperature, in the dark) and measured with a fluorescence spectrophotometer (excitation 411 nm, emission 482 nm, slit size 5 nm, 600 V).

### FLUORESCENCE *IN SITU* HYBRIDIZATION

Biomass was harvested from 20 ml reactor material by centrifugation (10 min 10,000 × *g*) and fixed for FISH analyses by addition of 4% w/v paraformaldehyde, incubating on ice (2 h), centrifuging (15 min 10,000 × *g*) and washing the resulting pellet with phosphate buffered saline (PBS, pH 7.2) and finally adding PBS and 100% EtOH (1:1) to reach a volume of 10% of the original sample. Fixed material was stored at -20°C until analysis. FISH analyses on fixed biomass from the start, after 1 and 6 months of the nitrite-fed period were performed as described by Amann et al. (1990), using 10 μl fixed material per hybridization. Vectashield (Vector Laboratories, Inc., Burlingame, CA, USA) mounting medium with DAPI (4,6-diamidino-2-phenylindole) was used to enhance the fluorescent signal and stain all DNA. Specifications and details of probes used in this study are presented in **Table 1**. Probes were purchased as Cy-3, Cy-5, and 5(6)-carboxyfluorescein-*N*-hydroxysuccinimide ester (FLUOS) labeled derivatives from Thermohybaid (Ulm, Germany). To visualize *Nitrosomonas* AOB and *Nitrospira* NOB simultaneously, probes NEU 653 (FLUOS) and NTSPA 712 (Cy3) were used together with their respective competitors (competitor probes consisted of unlabeled oligonucleotides) in single hybridizations at a formamide concentration of 35%. To detect *Nitrospina *sp. NOB, hybridizations were performed at 20% formamide concentration with probe NTSPN693. To stain all bacteria, a mixture of probes EUB338, EUB338 II, and EUB338 III was used for all hybridizations. Microscopic inspections were performed at a 1000-fold magnification. For image acquisition a Zeiss Axioplan 2 epifluorescence microscope (Zeiss, Jena, Germany) was used with the standard software package (version 3.1). Abundance estimates of cells hybridizing with a particular probe were based on visual inspection of three randomly taken FISH microscopy pictures per hybridization.

**Table 1:** Oligonucleotide specifications.

Name	Used for	(5′–3′)	Position*	Target	Reference
EUB338	FISH	GCTGCCTCCCGTAGGAGT	338	Most Bacteria	Amann et al. (1990)
EUB338 II	FISH	GCAGCCACCCGTAGGTGT	338	Most Planctomycetales	Daims et al. (1999)
EUB338 III	FISH	GCTGCCACCCGTAGGTGT	338	Most Verrucomicrobiales	Daims et al. (1999)
NEU653	FISH	CCCCTCTGCTGCACTCTA	653	Most halophilic and halotolerant	Wagner et al. (1995)
Competitor NEU653	FISH	TTCCATCCCCCTCTGCCG		*Nitrosomonas* spp	
NTSPA712	FISH	CGCCTTCGCCACCGGCCTTCC	712	Most members of the phylum Nitrospirae	Daims et al. (2001)
Competitor NTSPA712	FISH	CGCCTTCGCCACCGGTGTTCC		Nitrospirae	
NTSPN693	FISH	TTCCCAATATCAACGCATTT	693	*Nitrospina gracilis*	Juretschko (2000)
616F	PCR	AGAGTTTGATYMTGGCTCAG	8	Bacteria	Juretschko et al. (1998)
630R	PCR	CAKAAAGGAGGTGATCC	1529	Bacteria	Juretschko et al. (1998)
NTSPA1158R	PCR	CCCGTTMTCCTGGGCAGT	1158	Most *Nitrospira*	Maixner et al. (2006)
NSE87F	PCR	AGTGGCGAACGGGTGAGGAATA	87	Most *Nitrospira*	This study
NSE1124R	PCR	TCTCTCCAGAGTGCCCGGCATGA	1124	Most *Nitrospira*	This study
610IIF	Sequencing	GTGCCAGCAGCCGCGGT	479	Most bacteria	Ustinova et al. (2001)
M13F	Sequencing	GTAAAACGACGGCCAG	Region flanking cloning site	pGEMT easy vector	–
M13R	Sequencing	CAGGAAACAGCTATGA	Region flanking cloning site	pGEMT easy vector	–

* *E. coli* numbering.

### EXTRACTION HIGH MOLECULAR WEIGHT DNA

Biomass was harvested from 20 ml reactor content by centrifugation (20 min, 2400 × *g*) after 5.5 months with ammonia, and after 6 months with nitrite as the sole substrate, respectively. Biomass was also harvested from 50 ml reactor content after 12 months with nitrite as the substrate. High molecular weight DNA was extracted using a cetyltrimethyl-ammoniumbromide (CTAB) and sodium dodecyl sulfate (SDS)-lysis-based method adapted from Zhou et al. (1996). Biomass was suspended and incubated for 30 min at 37°C in a mixture of 675 μl CTAB extraction buffer (1g/100 ml CTAB, 100 mM Tris, 100 mM EDTA, 100 mM sodium phosphate, 1.5 M NaCl, pH 8), 50 μl lysozyme (10 mg/ml, 66200 U/mg) and 30 μl Rnase A (10 mg/ml, ≥5000 U/mg). After addition of 50 μl of proteinase K (10 mg/ml, 20 U/mg) and incubation for 30 min at 37°C, the mixture was supplemented with 150 μl 10% SDS and incubated at 65°C for 2 h. DNA was recovered by phenol/chloroform extraction and isopropanol precipitation after which it was suspended in 40 μl ultrapure water (MilliQ, Millipore SA, Molsheim, France) and stored at 4°C until use.

### PCR REACTIONS, CLONING, SEQUENCING, AND SEQUENCE ANALYSES

Polymerase chain reaction reactions (30 cycles, followed by a final extension for 10 min at 72°C) were performed in a T gradient PCR apparatus (Whatman Biometra, Göttingen, Germany) using GoTaq^®^ Green Master Mix (Promega Benelux BV, Leiden, the Netherlands). A PCR cycle consisted of, 1 min at 95°C, 1 min at annealing temperature (Ta) and 1.5 min at 72°C. For each 25 μl volume PCR reaction, 1 μl of 10-fold diluted high molecular weight DNA was used as the template. Resultant products were cloned using the pGEM-T easy vector cloning kit (Promega Benelux BV, Leiden, the Netherlands). Plasmid DNA was extracted using the GeneJET Plasmid Miniprep Kit (Fermentas GMBH, St. Leon-Rot, Germany). Clones were checked by restriction analysis of plasmid DNA (EcoR1, Fermentas GMBH, St. Leon-Rot, Germany). Sequencing (Sanger method) was performed at the division DNA diagnostics of the Human Genetics department of the University Medical Centre Nijmegen St Radboud. The ContigExpress program of the Vector NTI Suite 7.0 software package (InforMax) was used to assemble full-length clone sequences. Cloned 16S rRNA gene sequences were compared with their closest relatives in the GenBank database by BLASTN searches^1^. Phylogenetic and molecular evolutionary analyses were conducted using MEGA version 4 (Tamura et al., 2007). The Ribosomal Database Project (RDP) Classifier tool^2^ (RDP Naïve Bayesian rRNA classifier version 2.5, May 2012, RDP 16S rRNA training set 9) was used to evaluate the taxonomic position of sequences (Wang et al., 2007). Pairwise analyses to determine sequence identities were performed using the internet tool from the Georgetown University Medical Center^3^.

### 16S rRNA GENE SEQUENCE ANALYSES OF THE ENRICHMENT WITH AMMONIA AS THE SUBSTRATE

General bacterial primers 616F and 630R (Ta 56°C, all primer details are listed in **Table 1**) were used to amplify bacterial 16S rRNA gene sequences from DNA extracted after 5.5 months of operation with ammonia as the sole substrate. The resultant product was cloned, plasmids isolated and sequencing performed on 20 clones using sequencing primer 610IIF. For six clones the entire insert sequence was derived by additional sequencing reactions with primers M13F and M13R.

### 16S rRNA GENE SEQUENCE ANALYSES OF THE ENRICHMENT WITH NITRITE AS THE SUBSTRATE

After 6 months of operation with nitrite as the substrate, *Nitrospira*-targeted PCR (primer pair 616F and NTSPA1158R, Ta: 56°C) was performed on extracted DNA and the resulting product cloned. Eight clones (clones AC1-8) were randomly picked and sequenced with primer M13F. Additional sequencing was performed with primer M13R to obtain the full insert sequence of clone AC6.

### ACCESSION NUMBERS

16S rRNA gene sequences are available from GenBank under the following accession numbers: KC706457-706479. For sequences sharing at least 99% sequence identity (see “Results”) a representative sequence has been submitted. For the North Sea nitrifier enrichment cultures clone Cb9 (KC706457) represents the *Nitrosomonas *sp., Cb12 (KC706458) the *Nitrospina *sp. and Cb 18 (KC706459) “Candidatus *Nitrospira salsa*.” For the North Sea time series sequences clone P3_4 represents the cluster of 15 *Nitrospira *sequences.

### METAGENOME SEQUENCING AND RECONSTRUCTION OF THE 16S rRNA GENE SEQUENCE OF THE DOMINANT NOB

DNA extraction performed on 50 ml reactor biomass at the end of the incubation with nitrite as the sole substrate yielded 15 μg DNA based on spectrophotometric estimation using NanoDrop technology (Thermoscientific, USA). Eight microgram was subsequently used for pyrosequencing using the Roche 454 GS FLX Titanium sequencer (Roche, Switzerland) at the Department of Human Genetics Nijmegen at the Center for Molecular Life Sciences, Institute for Genetic and Metabolic Disease of the Radboud University Nijmegen Medical Center. To estimate *Nitrospira *abundance, all generated reads were mapped using CLC Bio Genomics Workbench (version 5.5.1) to a custom 16S rRNA gene sequence database which consisted of all unaligned sequences in release 10.29 (2,320,464 sequences) of the RDP 16S rRNA database (Cole et al., 2009), from which all sequences containing “uncultured” or “unidentified” in the description were removed. The resultant database (available upon request) contained 339,774 16S rRNA gene sequences. Through mapping (cutoff 90% identity over 90% of the read length) of the sequence reads on this database *Nitrospira *sp. 16S rRNA gene sequence reads were identified. The 16S rRNA gene sequence of the dominant *Nitrospira* NOB was reconstructed through a *de novo *assembly of those reads using the CLC genomics workbench.

### TRANSMISSION ELECTRON MICROSCOPY

To investigate the cell morphology of the enriched NOB using TEM, biomass harvested from 100 ml reactor content (by centrifugation for 20 min., 2400 × *g*) after 8 months of operation with nitrite as the sole substrate was taken. Cryofixation was performed by high pressure freezing and was followed by freeze-substitution in acetone containing 2% osmium tetroxide, 0.2% uranyl acetate, and 1% water, embedding in Epon resin and sectioning using an ultramicrotome for TEM analysis. Sample preparation was performed as described previously by van Niftrik et al. (2008).

### DETECTION OF THE ENRICHED *NITROSPIRA* NOB IN COASTAL NORTH SEA WATER

To verify that the enriched *Nitrospira* originated from the North Sea and was not a contaminant from our laboratory, high molecular weight DNA samples from a North Sea time series (Wuchter et al., 2006; Pitcher et al., 2011) were screened for the presence of *Nitrospira *by PCR analyses. A new primer pair perfectly matching the full-length 16S RNA sequence of the enriched *Nitrospira*, was designed (primers NSE87F and NSE1124R). These primers were tested in PCR reactions (Ta: 60°C) using the DNA extracted from the enrichment after 6 months of operation with nitrite as the substrate as a template. In addition, test reactions were performed using high molecular weight DNA extracted from *Nitrospira defluvii* and *Nitrospira moscoviensis* cells and plasmid DNA’s containing partial (1073 nt) 16S rRNA gene sequences from *Nitrospira* sublineages I (*Nitrospira defluvii*-like, 2 plasmids), II (*N. moscoviensis* -like, 2 plasmids), and IV (*N. marina*-like, 2 plasmids). To screen the North Sea time series, six pools were prepared from partial aliquots (3 μl of each sample) of the high molecular weight DNA samples from the time series (see **Table 2** in the Results). Prior to amplification, 5 μl of each pool was purified by excision of DNA-containing bands from low-melting point agarose gel (Electran wide range, low melting agarose, VWR BDH Prolabo) after electrophoresis to remove substances possibly interfering with PCR amplification. PCR products were cloned, and for 25 clones (3–5 clones picked per pool) plasmid DNA was extracted and sequencing performed with primers M13F and M13R.

**Table 2:** North Sea time series high molecular weight DNA samples.

Sample pool*	Time series samples	
P1	Oct–Nov–Dec 2003:	20-Oct, 27-Oct, 03-Nov, 13-Nov, 17-Nov, 25-Nov, 01-Dec, 12-Dec, 29-Dec
P2	Jan–Feb–Mar 2004:	02-Jan, 14-Jan, 30-Jan, 09-Feb, 15-Feb, 16-Feb, 23-Feb, 01-Mar, 08-Mar, 15-Mar, 22-Mar, 29-Mar
P3	Apr–May 2004:	13-Apr, 21-Apr, 18-May
P4	Aug–Sep 2004:	02-Aug, 09-Aug, 19-Aug, 23-Aug, 16-Sep, 27-Sep
P5	Oct–Nov–Dec 2004:	19-Oct, 08-Nov, 15-Nov, 29-Nov, 06-Dec, 16-Dec, 23-Dec
P6	Jan 2005:	14-Jan, 25-Jan, 31-Jan

* Pools consisted of a mixture of 3 μl of each time series sample.

## RESULTS

### NORTH SEA AOB AND NOB ENRICHMENT WITH AMMONIA AS THE SUBSTRATE

After a lag phase of 10 days, microbial ammonia oxidizers became active in the enrichment with ammonia as the substrate. Within 9 days, 500 μM ${NO}_{2}^{-}$ was produced from 500 μM ${NH}_{4}^{+}$. A second aliquot of 400 μM ammonium induced further nitrite accumulation at a higher rate (400 μM within 2 days) which indicates growth of ammonia oxidizers. After adopting a continuous mode of operation (*D* = 0.25 per day) to avoid nitrite toxicity, all supplied ammonium (750 μM) was converted to nitrite in a 1:1 ratio up to 3 months of operation. Hereafter, the nitrite concentration dropped to zero within a 14 day period indicating a rapid increase in nitrite oxidizer activity. During the subsequent stepwise increase of the influent NH_4_Cl concentration to 3 mM (after 4.5 months of operation), ${NH}_{4}^{+}$ as well as ${NO}_{2}^{-}$ reactor concentrations remained zero indicating complete consumption of both nitrogen species and therefore an active co-culture of ammonia and nitrite oxidizers converting 0.75 mmol of nitrogen per liter per day. The raise to 10 mM NH_4_Cl (after 5.5 months of operation) resulted in an ammonium and nitrite accumulation to final concentrations (at 6 months of operation) of 1.8 mM and 100 μM, respectively. During the last half month of operation therefore approximately 2 mmol of nitrogen were consumed per liter per day.

**FIGURE 1:**
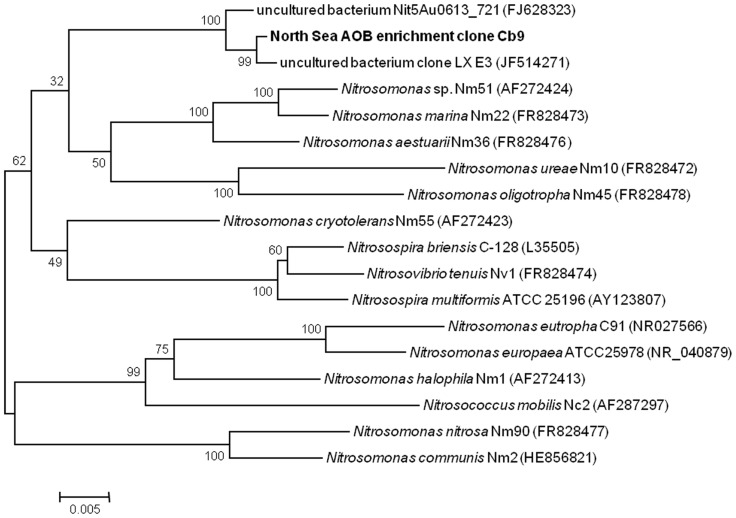
**16S rRNA gene sequence based phylogenetic tree showing the position of the enriched North Sea AOB (bold) within the betaproteobacterial AOB**. This unrooted bootstrap (1000 replicates) consensus tree was inferred using the neighbor-joining algorithm. Total of 1365 nucleotides were considered in the alignment. Bootstrap values are shown at the internal nodes. The scale bar is in the unit of the number of base substitutions per site.

The PCR performed with general bacterial primers on DNA extracted from biomass after 5.5 months of operation with ammonia as the substrate yielded correct-sized inserts (1500 nt). The 20 clones picked for plasmid isolation yielded 20 partial (695–845 nt) 16S rRNA gene sequences of which six contained recognizable nitrifier 16S rRNA sequences based on BlastN searches of the National Center for Biotechnology Information (NCBI) database and taxonomic assignment using the Classifier tool of the RDP. Taxonomic assignment of the remaining sequences resulted in five sequences assigned to unclassified bacteria, two to unclassified Planctomycetes, three to the genus *Phycisphaera* within the Planctomycetes, two to unclassified α-proteobacteria, one to the genus *Phaeobacter* within the α-proteobacteria, and one to unclassified Anaerolineae within the Chloroflexi. Nearly full-length 16S rRNA gene sequences generated from the clones containing a recognizable nitrifier sequence resulted in three *Nitrosomonas* (AOB) sequences (clones Cb9, 10, and 15; >99% shared sequence identity), two *Nitrospina* (NOB) sequences (clones Cb12 and 16; 99.5% shared sequence identity), and one *Nitrospira *(NOB) sequence (clone Cb18). The phylogenetic position of the putative *Nitrosomonas*-like AOB is shown in **Figure 1** which illustrates that the sequence from the enrichment culture is related to *Nitrosomonas marina* but does not cluster closely to any cultivated *Nitrosomonas *species. The closest match in the NCBI database (96% sequence identity) with a cultivated species was the 16S rRNA gene sequence of *Nitrosomonas *sp. **NM51 (Purkhold et al., 2000). This implies that the enriched AOB may represent a previously uncultured *Nitrosomonas *species. The closest match in the NCBI database (99% sequence identity) was to an unpublished marine clone sequence (JF514271, clone LXE3). For another sequence (FJ628323, clone NitA40631, Schmidtova et al., 2009) sharing 99% sequence identity to the sequence of the enriched North Sea AOB it was known that this sequence was retrieved from brackish water from the anoxic fjord Nitinat Lake, which is an environment with an ammonium concentration between 20 and 200 μM. The *Nitrospina *(NOB) sequences shared only 92% sequence identity to the 16S rRNA gene sequence of the cultivated species *Nitrospina gracilis *strain 3/211 (FR865038). The next taxonomically described match for the *Nitrospira* NOB clone sequence was 94% sequence identity with the 16S rRNA sequence of *Nitrospira marina *strain Nb-295 (X82559, Ehrich et al., 1995).

**FIGURE 2:**
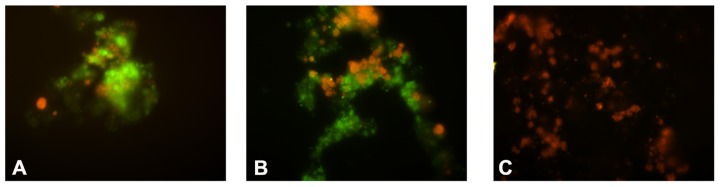
**Fluorescence microscopy pictures of the abundance of *Nitrosomonas* AOB and *Nitrospira *NOB during the NOB enrichment**. In green: cell hybridizing with probe NEU653 (targeting most halophilic and halotolerant *Nitrosomonas* spp.). In red: cells hybridizing with probe NTSPA712 (targeting most members of the phylum Nitrospirae). **(A)** Biomass after 1 month; **(B)** after 3 months; **(C)** after 6 months of operation with nitrite as the only substrate.

The FISH analyses revealed that the biomass at the end of the ammonium-fed enrichment (**Figure 2A**) consisted mainly (approximately 80% of the total population) of bacteria hybridizing with probe NEU 653. This indicates dominance of halotolerant/halophilic *Nitrosomonas*-like AOB. In addition, around 10% of the bacterial population hybridized with probe NTSPA712 indicating the presence of *Nitrospira*-like NOB. No hybridization with probe NTSPN693 was observed which suggests that *Nitrospina *NOB were a minority within the nitrifier community.

### NORTH SEA NOB ENRICHMENT WITH NITRITE AS THE SUBSTRATE

Within the first month of the nitrite-fed period (manual supply nitrite to 0.5–1 mM final concentrations) an average nitrite consumption rate of 1 mmol per liter per day was observed. Fourfold dilutions of the biomass, after 1 week and after 1 month, respectively, did not result in any observed change in nitrite consumption rate. During the operation in a fed-batch mode (between 1 and 11 months of operation) with stepwise increasing influent nitrite concentration (from 10 to 80 mM) followed by continuous operation (*D* = 0.05 per day; last month of operation) the nitrite consumption rate increased to a final value of 3 mmol per liter per day. The fourfold dilutions after 2 and 4 months of operation, again did not affect the observed nitrite consumption rate. The FISH analyses (**Figure 2**) of biomass after 1 and 6 months of operation with nitrite as the substrate revealed an increase in *Nitrospira* NOB (to a final ~80% of the total bacterial population) and decline in *Nitrosomonas* AOB (~1%), indicating that the population became dominated by *Nitrospira* NOB.

**FIGURE 3:**
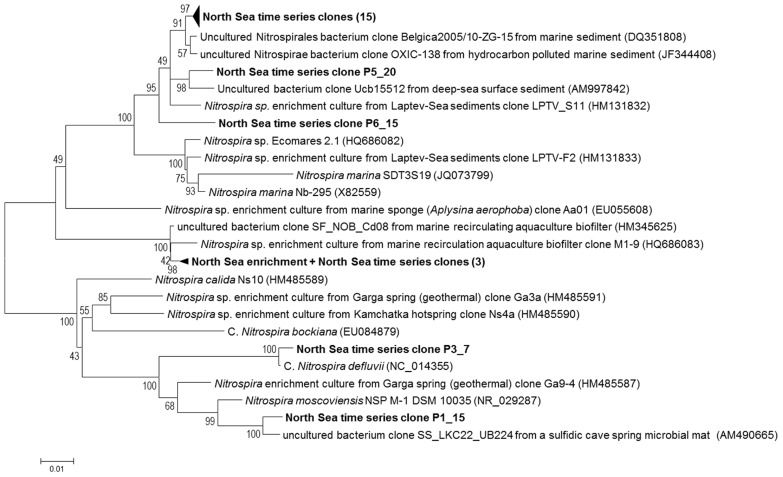
**Phylogenetic tree showing the positions of the enriched North Sea NOB and North Sea time series clone sequences (bold) in relation to known *Nitrospira* sequences and related sequences from the NCBI database**. This unrooted bootstrap (1000 replicates) consensus tree was inferred using the neighbor-joining algorithm. Total of 1013 nucleotides were considered in the alignment. Bootstrap values are shown at the internal nodes. The scale bar is in the unit of the number of base substitutions per site. For the North Sea time series clones the first number after the P indicates the pool (see **Table 2**) the clone was derived from. The second number identifies from which clone within the pool the sequence was derived. The 15 clone sequences within the upper collapsed branch are: P1_12, P1_27, P2_3, P2_17, P2_27, P2_46, P3_30, P4_33, P5_1, P5_3, P5_5, P6_8, P6_11, P6_12, and P6_14. The three North Sea time series clone sequences within the collapsed branch with the North Sea NOB enrichment sequence are: P3_4, P3_5, and P4_29.

The eight sequenced clones (AC1-8) obtained through PCR with primers 616F0 and NTSPA1158R using DNA extracted after 6 months of operation, contained 99% identical inserts based on pairwise alignment. Therefore the fully sequenced insert of clone AC6 was used as a representative for phylogenetic analysis (**Figure 3**). When looking at 16S rRNA gene sequences of taxonomically described species, the enriched North Sea *Nitrospira* is phylogenetically most related (94% identity) to *Nitrospira marina* strain Nb-295 (X82559, **Figure 3**). This analysis indicates that the enriched North Sea *Nitrospira* represents a new species for which the name “Candidatus *Nitrospira salsa*” (“salsa” = “salty”) is proposed. The closest relatives (>98.7% 16S rRNA gene sequence identity) of the enriched North Sea *Nitrospira* were bacteria from a biofilters of marine recirculating aquaculture systems (**Figure 3**, HM345625 and HQ686083). Sequence HM345625 is a clone sequence (clone SF_NOB_Cd08) derived directly from biofilter material (Brown et al., 2013). Sequence HQ686083, however, originated from an enrichment culture (M1 marine) derived from marine recirculation aquaculture system biofilter carrier material (Keuter et al., 2011). The nearly identical 16S rRNA gene sequences of the enriched North Sea *Nitrospira *and the marine aquaculture biofilter species indicate these are the same species.

The 16S rRNA gene sequence of clone AC6 is 99.9% identical to that of clone Cb18 based on pairwise analysis indicating that the *Nitrospira* NOB species represented by this clone was already present in the reactor prior to the switch to nitrite as a substrate.

### METAGENOME SEQUENCING AND RECONSTRUCTION OF THE 16S rRNA GENE SEQUENCE OF THE DOMINANT NOB

The 454 sequencing run on DNA extracted from biomass at the end of the incubation with nitrite as the sole substrate (after 12 months) generated, after quality trimming, 1,216,565 single reads with an average length of 405 nt. The mapping of all reads to the custom 16S rRNA gene sequence database resulted in 198 mapped reads, of which 147 mapped to *Nitrospira *sp. 16S rRNA gene sequences. This implies an abundance of *Nitrospira *sp. **16S rRNA genes within the total population of 74% which is in agreement with the 80% abundance estimated from the FISH analysis after 6 months of operation with nitrite as the substrate. The 16S rRNA gene sequence of the dominant *Nitrospira* NOB reconstructed from the 147 *Nitrospira* sp. **reads exhibited 99.9% sequence identity to the earlier obtained (clone Cb18 and AC6) sequences resulting from PCR analysis, suggesting that the same species persisted as the dominant NOB within the reactor.

### TEM ANALYSIS OF THE ENRICHED NORTH SEA *Nitrospira sp.*

**FIGURE 4:**
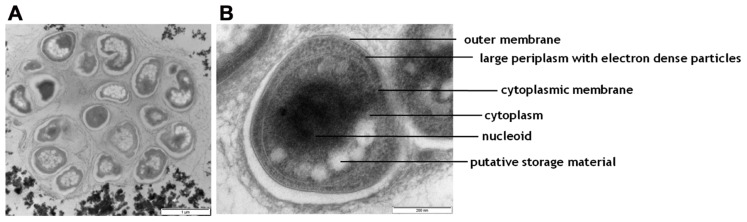
**Transmission electron micrographs of cells from the NOB enrichment**. **(A)** an overview picture of a representative aggregate of cells; **(B)** one representative cell with morphological features indicated.

The biomass was mainly situated in small aggregates in the culture. This was reflected in the electron microscopy pictures generated with the TEM analysis of the biomass, harvested from the enrichment after 8 months. These showed dense clumps of cells seemingly embedded in extracellular material (**Figure 4A**). Some typical morphological features of a representative cell are pointed out in **Figure 4B**. Most striking is the large periplasmic space containing many electron dense particles. In addition, large electron light particles are visible in the cytoplasm.

### DETECTION OF THE ENRICHED *Nitrospira* NOB IN COASTAL NORTH SEA WATER

*Nitrospira*-targeted primer pair 616F/NTSPA1158R did not yield significant amplicons (data not shown) for DNA samples from the North Sea time series, and therefore primers NSE87F and NSE1124R were developed. This primer pair, designed to specifically target the enriched “Candidatus *Nitrospira salsa*”, yielded correct-sized (1073 nt) amplicons with all tested templates (high molecular weight DNA from the North Sea enrichment, *Nitrospira defluvii* and *N. moscoviensis* as well as plasmid DNA from respectively sublineage I (*Nitrospira defluvii*-like), II (*N. moscoviensis*-like), and IV (*N. marina*-like). This demonstrates that this primer pair functions well for all tested *Nitrospira* species and does not specifically target the enriched North Sea species.

Screening of the six separate pools (see **Table 2**) from the North Sea time series of high molecular weight DNA samples with this primer pair resulted in 25 16S rRNA gene clone sequences of which 22 contained a *Nitrospira *sequence. Three (clones P3_4, P3_5 and P4_29) clones exhibited a sequence identity of 99% to the 16S rRNA gene sequences of “Candidatus *Nitrospira salsa*” (**Figure 3**). The majority (17) of the *Nitrospira* 16S rRNA gene clone sequences retrieved from the North Sea time series, however, were most closely related (97–99% sequence identity) to a clone sequence (DQ351808, clone Belgica2005/10_ZG-15) retrieved from marine sediment (Gillan and Pernet, 2007) and shared only 91–92% sequence identity with the 16S rRNA gene sequence of “Candidatus Nitrospira salsa”.

Surprisingly, North Sea time series clone P3_7 contained an insert most resembling the 16S rRNA gene sequence of *Nitrospira defluvii* (99.5% sequence identity to sequence NC_014355, Lücker et al., 2010) and clone P1_15 an insert resembling the 16S rRNA gene sequence of *Nitrospira moscoviensis* (97% sequence identity to NR_029287, Ehrich et al., 1995), which are *Nitrospira *species associated with freshwater environments.

## DISCUSSION

### BIOREACTOR CULTIVATION OF MARINE NORTH SEA NITRIFIERS

The bioreactor approach adopted to enrich North Sea nitrifiers proved successful. Within 6 months a marine assemblage of AOB and NOB was obtained by means of cultivation with ammonia as the substrate. The results from 16S rRNA gene-targeted PCR followed by cloning and sequencing indicated the presence of putative *Nitrosomonas*-like AOB (3/20 clones), *Nitrospira*-like NOB (1/20 clones), and *Nitrospina*-like NOB (2/20 clones), representing novel species. The FISH analysis demonstrated the abundance of the *Nitrosomonas*-like AOB (80% of the total bacterial population) and *Nitrospira*-like NOB (10%) of the total bacterial population, but failed to detect *Nitrospina* cells. Based on these results *Nitrosomonas* AOB and *Nitrospira *NOB are assumed responsible for the observed conversion of 2 mmol of ammonium per liter per day. Switching to nitrite as the sole substrate resulted in a high enrichment (80% of the total population based on FISH analysis) of *Nitrospira* NOB within another 6 months. The *Nitrospira *16S rRNA gene sequences obtained from this point in time proved identical to the sequence obtained from the AOB/NOB co-culture indicating the species originally present in the marine assemblage was successfully stimulated. Phylogenetic analysis showed the enriched North Sea *Nitrospira* represents a novel species (“Candidatus *Nitrospira salsa*”) only distantly related (94% 16S rRNA gene sequence identity) to the next taxonomically described species *Nitrospira marina*. In the study by Keuter et al. (2011) the *Nitrospira *in marine enrichment M1, derived from a marine recirculation aquaculture system, is hypothesized to originate from North Sea water because the system was started and refreshed with North Sea water. The high (within species-range) 16S rRNA gene sequence identity (>98.7%) of “Candidatus *Nitrospira salsa*” to the 16S rRNA sequence retrieved from enrichment M1 corroborates this.

The 16S rRNA gene reads mapping approach and subsequent 16S rRNA gene reconstruction based on metagenomic data corroborated (74% of all 16S rRNA gene reads were affiliated with *Nitrospira*) the FISH abundance data and showed that the enriched North Sea *Nitrospira *sp. persisted in the culture converting 3 mmol of nitrite per liter per day after 12 months of operation suggesting a quite stable microbial community. The TEM analysis data fits well with the known cell morphology of *Nitrospira *species*.* Watson et al. (1986) reported the presence of a large periplasmic space for *Nitrospira marina* and the presence of glycogen and polyphosphate deposits in cultures and Spieck et al. (1998) identified electron dense particles from the periplasmic space of *Nitrospira moscoviensis* as the nitrite-oxidizing enzyme system. In accordance with the aforementioned, the most striking attribute of the enriched North Sea *Nitrospira* sp. was a large periplasmic space containing putative proteins of the nitrite-oxidizing enzyme system as suggested by the presence of many electron dense particles. In addition, the cytoplasm contained putative storage material, such as glycogen, visible as large electron light particles. Most likely, storage of carbon was triggered by a phosphate limitation during the cultivation. This because TEM analysis was performed on biomass after 8 months of operation and to prevent carbon limitation the reactor had been supplemented with additional CO_2_ from month 7.5 onward. Additional phosphate to prevent phosphate limitation was provided only after 8 months which may have led to an imbalanced situation in which carbon was plentiful but phosphate was limiting.

### RELEVANCE OF THE ENRICHED NITRIFIERS IN DUTCH COASTAL NORTH SEA WATER

It has been reported by Pommerening-Röser et al. (1996) that affinity for ammonia varies among members of different lineages within the AOB genus *Nitrosomonas* but tends to be relatively similar within a specific lineage. The clustering of the enriched North Sea *Nitrosomonas* AOB 16S rRNA gene sequence with *Nitrosomonas *species commonly associated with low substrate environments (**Figure 1**) therefore suggests this species is likewise adapted to relatively low substrate conditions. This thought is strengthened by the origin of clone sequence FJ628323 (clone NitA40631) which shares 99% sequence identity to the sequence of the enriched North Sea *Nitrosomonas* AOB. This clone sequence was retrieved from brackish water from an anoxic fjord Nitinat Lake (Schmidtova et al., 2009), for which an ammonium concentration between 20 and 200 μM was reported. Maximum ammonium concentrations in the coastal North Sea water from which the enrichment is derived have, however, been reported to range from 10 to 13 μM during the winter months (Pitcher et al., 2011) which implies aerobic ammonia oxidation will likely be catalyzed by microorganisms with even higher affinities for ammonia. Moreover, AOB have been shown to be outnumbered by AOA (based on 16S rRNA and *amo*A gene copy numbers) in time series of Dutch coastal North Sea water (Wuchter et al., 2006; Pitcher et al., 2011). The enriched North Sea *Nitrosomonas* AOB therefore might exhibit a low abundance in coastal North Sea water and its contribution to *in situ* nitrification may be minor. The retrieval of clone sequences (3/20) from the North Sea time series nearly identical to the 16S rRNA gene sequence of “Candidatus *Nitrospira salsa*” proves that this species does occur in the North Sea. The higher abundance of clone sequences (17/22) forming a separate distinct cluster suggests that another *Nitrospira* species may actually be more abundant and potentially contribute more to *in situ* nitrification. Moreover, our data suggested that a minor portion of the nitrifier community may have consisted of *Nitrospina* NOB after 5.5 months of enrichment with ammonium. *Nitrospina *species have often been detected in marine environments (e.g., Mincer et al., 2007; Beman et al., 2010; Santoro et al., 2010; Füssel et al., 2011). Based on our present study, we cannot exclude that *Nitrospina* NOB may be present in greater abundance or contributing to a greater extent to *in situ* nitrite oxidation. Cultivation in a bioreactor set-up offers a higher degree of control over environmental parameters (pH, T, substrate concentration, product concentration) than more traditional batch cultivation. Selection for a particular species due to the cultivation conditions, however, cannot be excluded completely. Our enrichment was performed with a maximum nitrite concentration of 750 μM (during the first month of operation). Off et al. (2010) reported nitrite tolerances for different species of *Nitrospira *NOB ranging from a low 1.5 mM for enrichment culture Aa01 derived from a marine sponge to intermediate (6 mM) for *Nitrospira marina* to high (15–25 mM) for freshwater *Nitrospira *species. Based on this, the present bioreactor cultivation would be expected to yield a *Nitrospira *NOB associated with low levels of nitrite. Indeed the closest relatives (based on 16S rRNA gene sequence analysis) of the enriched North Sea *Nitrospira* originated from recirculation aquaculture systems which were described as relatively low nitrite environments (10–40 μM nitrite reported by Keuter et al., 2011; nitrite below detection reported by Brown et al., 2013). Substrate concentration may have contributed to the dominance of “Candidatus *Nitrospira salsa*” in the final enrichment instead of *Nitrospira marina*-like or *Nitrospina*-like NOB.

Strikingly, the only pools (P3 and P4) from which “Candidatus *Nitrospira salsa*” sequences were derived were also the only pools consisting of samples from outside the winter months (spring and summer; **Table 2**). *Nitrospira defluvii* (enriched from wastewater treatment sludge, Spieck et al., 2006; Maixner et al., 2008; Lücker et al., 2010) and *Nitrospira moscoviensis *(isolated from a partially corroded area of an iron pipe of a heating system, Ehrich et al., 1995) are commonly associated with freshwater environments. **The detection of clone sequences most related (99.5% to *Nitrospira defluvii*, 97% identity to *Nitrospira moscoviensis*, respectively) to 16S rRNA gene sequences from these *Nitrospira* species may be caused by terrestrial input (e.g., riverine influx) at the sampling site.

### OUTLOOK

Further research, e.g., selective inhibition experiments of AOA versus AOB activity (Yan et al., 2012), may clarify the role of the enriched *Nitrosomonas *AOB species in Dutch coastal North Sea water nitrification. Bioreactor enrichments adopting more stringent substrate levels may result in marine microbial assemblages with a totally different species composition, which would be useful to compare and contrast to the one presently described. Collection of *in situ *abundance data (e.g., by quantitative PCR analyses) for different species of NOB (e.g., “Candidatus *Nitrospira salsa*” versus other *Nitrospira* sp. and *Nitrospina* sp.) may help identify which NOB are of relevance to *in situ* nitrification. Seasonality in the abundance of this species may be corroborated by future reactor or laboratory enrichment experiments performed at different temperatures. Screening of a high resolution time series may aid in elucidating temporal changes in NOB community composition. The availability of the new *Nitrospira *species enrichment culture facilitates further in-depth studies such as determination of physiological constraints and comparison to other NOB species. Such characterizations will increase our understanding of microbial nitrogen cycling.

## Conflict of Interest Statement

The authors declare that the research was conducted in the absence of any commercial or financial relationships that could be construed as a potential conflict of interest

## Footnotes

1 http://www.ncbi.nih.nlm.edu/BLAST

2 http://rdp.cme.msu.edu/classifier/classifier.jsp

3 http://pir.georgetown.edu/pirwww/search/pairwise.shtml
